# Making green growth a reality: Reconciling sobriety with stakeholders’ satisfaction

**DOI:** 10.1371/journal.pone.0284487

**Published:** 2023-08-23

**Authors:** Caroline Gans-Combe, Jae-Yun Jun, Waleed Mouhali, Yves Rakotondratsimba, Aïcha Baccar

**Affiliations:** 1 INSEEC Business School, OMNES Education, Paris, France; 2 ECE, OMNES Education, Paris, France; West Pomeranian University of Technology, POLAND

## Abstract

The notion of sobriety is considered a key variable in various energy transition scenarios. Often associated with a form of punitive ecology, it is, nevertheless, possible to make it a component that supports green growth, by linking it to the concept of "satisfaction". In this work, we have invented a way to achieve both “digital”, “economic”, and “ecological” sobriety, while ensuring the satisfaction of the end user. Directly correlated to the production of goods or services, the satisfaction function is built on the well-documented marginal utility function, which measures the need (or not) to consume further resources to satisfy the economic agents. Hence, it is justified and exists because it stands for the expectations of end users and makes sure the latter is met. This product itself is a function of the allocation of a set of resources, mapped using activity-based costing tools (ABC method). In this work, we focus on an AI proof-of-concept and demonstrate that it is possible to reach numerical sobriety by controlling the size of the training dataset while ensuring roughly the same model performance. In general, we show that it is possible to preserve the efficiency of AI processes while significantly minimizing the need for resources. In this sense, after establishing an analytical model, we suggest reducing the amount of data required to train the machine learning (ML) models, while guaranteeing zero change in terms of performance (say their accuracy). We show that it affects the energy consumed, and, thereby, the associated cost (i.e., economic and ecological) and the associated CO2eq emission. We thus confirm the existence of a "triangle of sobriety". It is defined as a virtual circle governed by a digital-economic-ecological sovereignty. We also propose that if AI production processes have a potential for sobriety, all identical activities have the same characteristics, thus opening the path to green growth.

## Introduction

The notion of sobriety (understood as frugality) is considered a key variable [1] and one of the pillars of the ecological transition in different energy transition scenarios. The appetite for growth and individual well-being is not a whim; it is rooted in the deepest part of the human psyche. It is the desire to benefit from what others enjoy, in other words, mimetic envy [2]. This envy, however inglorious or negatively connoted, remains a strong driving force of economic and social development, as demonstrated by [3] in his political-historical analysis of the phenomenon. Based on this premise, two facts can be explained concerning the current situation of the planet.

- The failure of general adherence to actions tends to reduce the possibilities of satisfying human desires (whatever they are, from energy to the consumption of goods). No one desires to be left wanting, or more precisely, countries, still on the road to development, do not see why they should have less ambition than their neighbors, who are already more advanced in this field. No one appreciates sacrifice, especially when it benefits others [4]. However, this forced asceticism remains the only path explored by policies to fight against global warming.
- It is observed that a suppression economy is both conceptually non-functional and inhuman—concerning incompatibility with the human psyche [5]—as it doesn’t show any kind of respect to mankind. Beyond coercion, adhesion is achieved through positive motivation [6]. Based on this postulate and the imperative of universal adherence to a reduced impact of human activity on the planet, what are the crossroads that are available to us, so that it becomes the reality for all? In other words, is it possible to make one adhere to consensual sobriety where forced sobriety is a severe challenge, if not an impossibility?

Hence, sobriety is currently understood as the need to apply consumption restrictions and changes in attitudes, essentially aimed at final consumers and citizens. However, this approach, while it may temporarily rely on the civic sense of economic actors, quickly encounters the Maslovian aspirations of every person, especially if the effort is not equitably shared, and some people are given special privileges [7]. Thus, an unequally shared burden of sobriety could lead to a rejection of the concept, which would not only be a disaster for the planet but would also generate a deep social crisis [8] that the climate and energy emergency must avoid at all costs.

In the current discourse, sobriety is thus synonymous with moderation without consideration for individual needs, however superficial they may seem to be [9]. It is a question of depriving oneself of the common good, which would bring the ethics of sobriety into direct conflict with human expectations, which are themselves based on our motivations [10]. Appetence would be the evil; asceticism the rule. Is this vision realistic? This remains to be explored, though it is likely that the failure of all attempts, at significant ecological moderation in recent years, lies in this reading since conviction is much more effective than constraint [11]. In any event, this paradigm shift has such impacts and requires such a consensus that it calls for a temporality that no one has today. This does not imply, however, that we should indulge in fatalism by falling into the trap of the twelve excuses delaying positive action [12], for the benefit of the climate.

Thus, in this context, we hypothesize that it is possible to define the notion of sobriety more robustly than as a simple behavioral change, notably by adding a component of preservation of operational outputs and growth. Sobriety in our view does not imply deterioration in production conditions or losses in quality of life. Thankfully, there is a body of thought, proposed by Max Weber, that theorized the intimate link between economic growth and sobriety through the observation of real situations at the beginning of the 20^th^ century [13].

With him, we posit that it is not growing as a creation of collective wealth that is called into question by the climate crisis, but rather how this growth is achieved: one built on resources waste and the illusion that these are available without any limitation. It would, therefore, be possible to preserve the promises of growth and the improvement of economic situations, for the benefit of all, through the accumulation of capital, while simultaneously being climatically virtuous".

i.e., by recasting the way we do things. This is nothing less than rehabilitating the ethics of capitalism [14]. As Foster et Holleman [15] reminded us, more than a decade ago, Weber had already spoken about the end of coal–the fossilized fuel. He was already thinking in terms of scarce resources and insisted on testing the viability of resource-constrained productive processes as a basic rule of pragmatic and structured economic practice, which was, after all, marked by Protestant circumspection. In his posthumous book Economy and Society, Weber refers to this vision as an integral part of his four rationalization processes, and the in-depth knowledge of the processes as the driving force of this rationality. On the way to unrestrained global growth, we have lost the ability to produce frugally [16]. The advantage of this approach, based on the control of productive components and processes, is that it allows us to protect ourselves from the possible loss of a resource, in a context where many scientists and others are beginning to sound the alarm about the risks posed by our society’s propensity to waste [17].

As this systemic risk is integrated into the production processes, it becomes possible—in the event of a tension or crisis—to substitute either the anticipated alternative where it exists or to propose a different production path for the time needed to stabilize the ecosystem [18]. Had Weber not been forgotten, the impact of the current energy crisis would likely have been largely mitigated by the existence of plans for substituting hydrocarbons in industrial processes, limiting tension in the markets, speculation, and the resulting inflation.

Both ecological and digital transitions (i.e., digitalization and decarbonization) are viewed as economic and industrial opportunities for today’s economies, as they have the potential to generate substantial co-benefits in terms of employment, competitiveness, and general well-being. Verdolini [19] has established several policy operational suggestions, which include the need to replace aging infrastructure, lessen reliance on energy, and sustain economic expansion.

Artificial Intelligence (AI) is a domain in which we can observe digital transitions, with great economic and industrial opportunities, having significant ecological impacts. Among many aspects addressed by AI, we are particularly interested in machine learning (ML) due to the unceasingly-made great advances in this domain. Machine Learning is a discipline of study that gives machines the ability to learn, based on mathematical and statistical approaches, that is, to improve their performance in solving tasks without being explicitly programmed. With the purpose to show that our argument given in the present work fits in a general framework, we implemented a well-known computer-vision algorithm based on a convolutional neural network (CNN) [20], and we made it learn to solve an image-classification problem using images from a well-known database (CIFAR-10 [21]).

Moreover, the current situation shows us that some strategic industries, such as AI [22], are more affected than others by the multiple crises (climate and energy) and the related issues that we experience. Current responses to these challenges tend to oppose, in some way, energy efficiency to performance [23]. We consider this unacceptable considering the industry’s promises for humanity through automation, smart decision-making, human error avoidance, and much more. The advances in AI may make possible a better human life. For these advances, better, more powerful, faster, and larger resources are increasingly demanded.

However, increasing the capacity required to improve computing devices (such as servers) and algorithms for processing continuously growing data may go against environmental preservation, causing climate change and escalating disaster risks. Many analyses and studies show that AI can harm the environment. As of 2018, the computation used in various AI training models has doubled every 3.4 months since 2012, representing a 300,000% increase [24].

As far as green AI is concerned, Strubell et al. [24] recommend that the researchers in this domain: 1) should report the training time and the sensitivity of hyperparameters, 2) need equitable access to computation resources, and 3) should prioritize computationally efficient hardware and algorithms [24].

On the other hand, Patterson et al. [25] established a model of 4Ms, to reduce energy consumption and carbon emissions, in the AI domain. These 4Ms are as follows: Model (for selecting efficient ML model architectures), Machine (for using processors optimized for Machine Learning (ML) training), Mechanization (for cloud computing), and Map (for choosing the geographical location with the cleanest energy). In addition, a framework for carbon-aware data centers has been designed involving energy investments, energy storage, and computation shifting [26]. Making judicious hardware-software choices such as “platform-level caching”, “GPU acceleration”, and “algorithmic optimization” could greatly optimize different language models. In Wu et al. [27], the environmental impact of the super-linear growth trends, for AI, is explored from a holistic perspective, spanning Data, Algorithms, and System Hardware by characterizing the carbon footprint of AI computing by examining the model development cycle across industry-scale machine learning use cases and, at the same time, considering the life cycle of the system hardware.

These studies mainly focus on reducing the energy waste and carbon emissions caused by AI, but, as we observe, the economic and societal aspect has not been much tackled. Moreover, the proposed solutions focus primarily on reducing the efficiency of industrial processes–including AI–as a necessary tradeoff for optimizing resource use [28]. As already mentioned, we do not subscribe to this approach.

In the present study, we have defined a new analytical and operational approach, based on sobriety, constrained by the satisfaction level defined for an AI process. It is an innovative methodology going beyond the carbon footprint indicators studies. We assume that it is more pertinent to find sobriety wells than the CO2 emission or (carbon pits). We consider that the carbon pits indicator is not sufficiently operational for economic stakeholders. Indeed, the CO2eq rate strongly depends on calculation methods (which are not consensual). Furthermore, this approach is limitedly usable when considering the economic aspect of an industrial process. This is even more in the case of AI processes.

Hence, after carefully defining what we mean by satisfaction, a multivariable mathematical function), we can define the triangle of sobriety as an intercorrelated view of digital, economic, and ecological sobriety.

This is a new paradigm to foresee environmental impact reduction. As we are interested here in demonstrating that it creates economic expenditure reduction, we couple our model with an economic cost model: where others look for carbon wells, we look for sobriety pits. That is why we assign ourselves the task of finding a way to achieve digital, economic, and ecological sobrieties, while maintaining end-user satisfaction, or, in other words, the operational efficiency of processes.

How should we approach end-user satisfaction? What is it? Economic theory offers three responses to this fundamental question: (1) utility, (2) in a cardinal or ordinal reading, or (3) the "choices" approach, which emphasizes purchasing decisions over satisfaction [29]. However, these three theoretical frameworks assume that end-users express contentment by arbitrating a price/product/quantity trade-off [30], which is partially observed, but not a unique decision point. A product’s originality may increase appetite, which is more essential than satisfaction.

Consumer satisfaction is related to utility, more specifically marginal utility, which estimates the change in utility produced by a one-unit change in consumption or use. The economists value marginal utility (the scarcity of goods) in particular. Market biases affect the utility function [31]. Neoclassical value is based on the usefulness of the most recently exchanged unit. Gossen’s first law [32] asserts that each unit consumed diminishes consumer demand. A good’s marginal usefulness is determined by its added consumption, which in the end determines its prices.

Pareto and Slutsky [33], through the ordinal approach, found that ordinal theorists underestimated utility quantification. They proposed the method of measuring utility by the end-perceived user’s importance, rather than numbers. Substantial rationality underpins this idea. Substantive rationality means people seek maximal enjoyment with the least resources, at the lowest possible price.

Both theories assume that a reasonable buyer should maximize satisfaction or utility. Thus, consumers can quantify their utility by using a good or service, using a precise quantitative index. This approach represents the preferences of economic agents between multiple options (baskets of goods/services, financial portfolios) over a potentially infinite period, where they all have the same information, derive satisfaction from their consumption (or use, we speak of positive utility), are limited in their purchasing capacity (they cannot borrow), and allocate all of their money to realizing this utility (e.g., arbitrage, investment, distribution). These preconceptions rarely match reality. This reading asks and attempts to answer the question: how does an economic actor divide their budget among accessible products and services?

Being aware of the shortcomings of this approach, economic theory has attempted to develop a "new theory of the consumer" [34] that emphasizes the existence of a consumption process, similar to the production process, which makes the consumer/end-user appear to act to achieve explicit ends, rather than in an impulsive/irrational manner, whose role is limited to "consuming" goods to satisfy his needs. This perspective gives domestic behavior the materiality and objectivity of productive behaviors and efficient judgments. However, it has concealed a second novel component of the new theory, which is providing a framework for studying subjective preference development. The classic theory of utility ignores this perspective, even though it is vital if the customer must identify his purposes and seek knowledge to arbitrate decisions [35]. Here, arbitrage matters because it affects the customer’s choice. This hypothesis states that consumers don’t differentiate between products/services if they perform the same services. Thus, in such a situation, the latter will accept both a waste-free and an environmentally harmful product. This is described by the indifference function.

Demonstrating a product or service’s capabilities can boost end-user approval [36] in maintaining contentment if the service supports it. The user is devoted to satisfaction, not a product [37].

Trade-offs are different views on the availability of goods/services and pricing as an attribution of value, all within a volumetric logic (a price corresponds to a given quantity). Maslovian logic progresses from one state of societal achievement to the next and from one product/service life cycle to the next, without addressing consumer happiness, that is the capacity created by the acquisition of a product or service to expand production to a higher level.

Hence, the crucial question behind this approach is: what does AI fully consume in terms of resources?

From an economic perspective, it is complex to put a price on AI because the pricing models for all AI-related resources do not exist, and not all its parameters are priced. A full AI costing model would enable the identification of the usage of inadequate resources (and related excess costs), as well as the possibility to optimize such resources while maintaining algorithmic efficiency within the parameters of a business plan. The transition from using carbon footprint to using economic cost as an indicator, in the effort to reduce emissions, will incentivize AI-oriented industries to adopt not only energy-efficient but also cost-efficient AI algorithms and infrastructure services in their AI business development journey, to make green growth a reality. In this work, we will demonstrate that it is possible to implement a method that helps to achieve “digital”, “economic”, and “ecological” sobrieties. In general, we are interested in investigating how the progress made in AI impacts the increase of the cost (that is needed for this progress) and climate change. Particularly, in this work, we are interested in analyzing the reduction of the data required to train the ML models (i.e., digital sobriety), while guaranteeing almost no change in terms of their performance (say their accuracy), affects the energy consumed, and, therefore, the associated cost (i.e., economic sobriety) and the related emission of CO2eq (i.e., ecological sobriety).

Precisely, at the heart of the effectiveness of AI systems, “accuracy” is understood as satisfaction (i.e., the response to an expectation or need) [38].

Extrapolating Maslow’s matrix [39] into a satisfaction matrix, we tend to determine at which point(s) (moment), in the literal and geometric sense of the term, the level of satisfaction of a stakeholder is sufficient to move on to the next one [level of satisfaction], but even more so, at which level of use of a given resource, the stakeholder considers itself satisfied and, therefore, turns its attention to another level of need to be satisfied.

**Fig 1:**
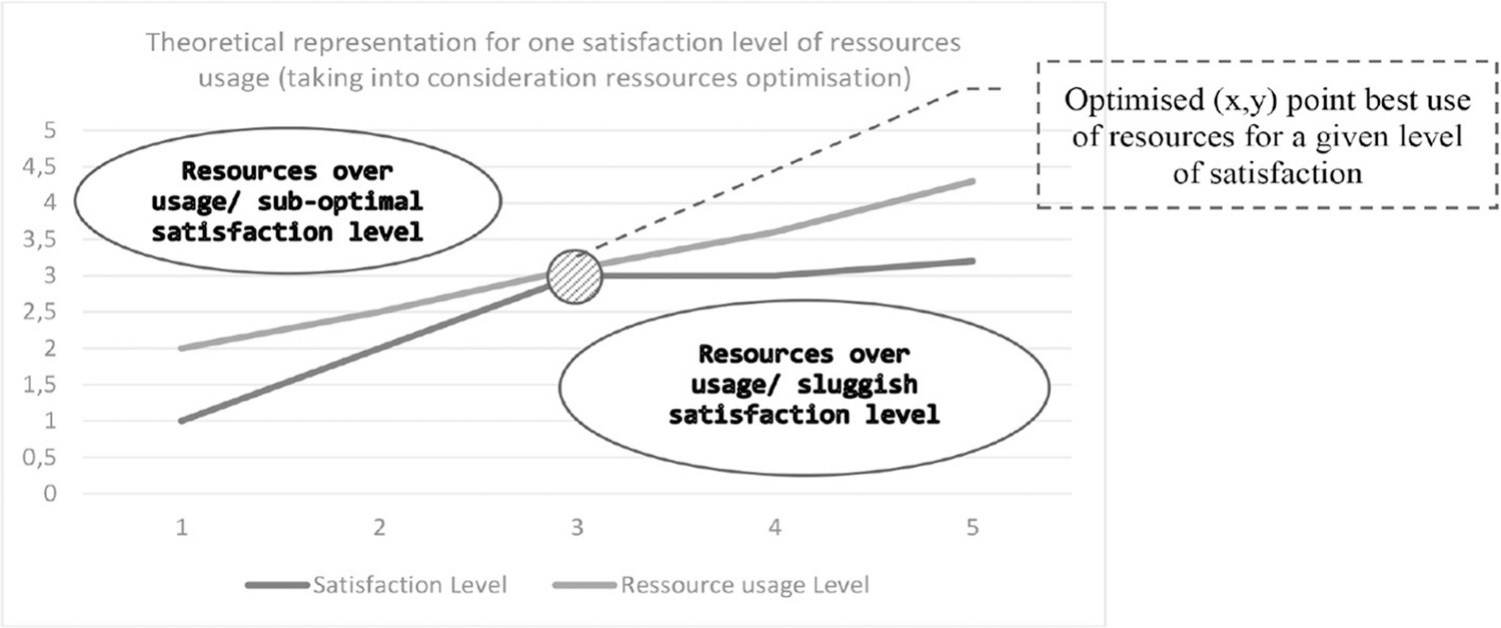
Theoretical representation of satisfaction.

Simply put, we have sought to establish a satisfaction/resource pair to calculate this precise transition point and to use the matrix pairs, thus created, to locate and develop the optimal satisfaction obtained for a given resource use (Fig 1).

Satisfaction, here, is achieved when the process delivers sufficient output to deal with the question put to it, without any margin of error other than a statistically acceptable one. An AI process will convert “resources” into “satisfaction”.

There is, therefore, a satisfaction function that meets this imperative. In this context, modeling sobriety turns out to be a challenge of real complexity. As such, satisfaction is directly correlated to the product resulting from the said system. Satisfaction itself is a function of the allocation of a set of resources, which we have mapped using activity-based costing (ABC) tools [40]. This mapping enables us to identify the different drivers describing the artificial intelligence activity and to test the effects of their variation on the accuracy and the expected deliverable. The ABC approach states that satisfaction is a function of tasks and resource allocation, through interdependencies and constraints, that require further analysis. We tested driver dependencies, demonstrating, for example, that energy consumption was a function of not only the algorithm used but also the volumes of data considered for training the learning system. By stressing the interlinkages and tensions, we discover that optimal accuracy is achieved when data consumption reached 68% and that further data processing does not raise precision.

The satisfaction approach, therefore, allows us to state that without changing the process, and by analyzing the interdependencies between drivers and resources, it is possible to identify sobriety pools and reduce the consumption of an AI system by 32%. In other words, AI systems have a potential sobriety of 32%. As this analytical approach is based on an analysis of homogeneous activity drivers, it should be portable beyond the present proof of concept [41].

We adopt the methodology, explained in the next section, to answer the above research questions. To validate this approach, we design a static test framework—derived from ex-post collected data—allowing us to establish parametric reference elements regarding the efficiency of greenness criteria and related arbitration. In this article, we limit our studies to this static framework. Hence, we propose an operational framework, allowing us to pose a model of sobriety, concerning the use of AI which will not impair or diminish performance or accuracy.

## Frame and methods

### ABC frame

The ABC (or activity-based costing) method can be defined as follows: cost objects (products, customers…) consume activities, which in turn, consume resources. It is the activities of the stakeholders that are fundamental and not the resources as such because the use of resources is directly correlated to the use made of them. This is a reversal of the theoretical approach to price formation and the associated trade-offs. This method of analyzing costs, by activity, was first used in large American industrial companies in the 1950s but was made public only in the 1980s and 1990s, through the work of Kaplan and Cooper [42]. The latter conducted several studies on the American market to show that there are both direct and indirect costs, which are not always known by companies. In 1992, they rejected the notion of fixed costs in favor of capacity costs. They proposed the following equation:

Costofavailablecapacities=costofusedcapacities+costofunusedcapacities

The ABC method was adopted based on this observation and its variations [43]. It is a setup to facilitate the calculation of the company’s costs in the given capacity utilization context. The objective of the ABC method is to model expenses, by activity, to better manage them. It allows analysis of the most profitable and the least profitable activity. The objective is, therefore, to identify the real cost factors and potential savings to improve either the profitability of either products and services or the customer approach, i.e., the minimization of the ratio of customer acquisition costs to the average basket. To do this, we establish a benchmark based on the Pareto principle (or the 20/80 law), comprising three distinct groups that will classify costs by activity in descending order:

✓ Group A: 20% of the products/services represent approximately 80% of the turnover.
✓ Group B: 30% of the following products represent about 15% of the turnover.
✓ Group C: 50% of the remaining items represent about 5% of the remaining turnover.

When performing an ABC analysis, it is of utmost importance to focus on the analysis of the references that generate 80% of your turnover. This vision clearly shows not only that half of the activity generates most of the turnover of a given structure, but also that the improvement of the profitability passes by a strong understanding of these first groups. In addition, it also indicates that the activities and resources used for the residual group, C, are not necessarily used wisely.

It is in these two analyses (the intuitive one relating to group C and the counterintuitive one relating to groups A and B) that the sources of savings for the company probably lie. Based on these hypotheses, we propose to move from the analysis by the profitability for the stakeholders to the analysis by their satisfaction.

As stated above, we start from two assumptions: (1) that the purchase of a good or service provides satisfaction to the user [44], and there is an expectation behind any act of purchase, and (2) that any activity undertaken to produce anything beyond this level of satisfaction constitutes a source of sobriety, since it is devoid of operational and economic justification. The challenge of this approach is to establish the saddle point or the matrix where satisfaction and sobriety meet, where for a given activity and production, the end user is satisfied, and this satisfaction will not increase, regardless of the mobilization of additional resources.

To do this, it is necessary to know in detail each production process and each activity related to this production process that we will define later by matrix points. These activities can range from marketing to convincing stakeholders to acquire something (goods or services), to reference management, RD, or order processing. Once this is done, it is necessary to identify the indirect expenses, that will be reprocessed, and link the expenses and the activity. We can see that there is a correlation between expenses and activities, which avoids the pitfalls of other pricing methods, in particular the arbitrary use of allocation keys, which is certainly standardized but often empirical [45].

The objective of the ABC method is to identify real cost factors and potential savings to improve the profitability of products and clients. To do this, it analyzes the company’s transversal processes by setting up indicators, called drivers, allowing us to highlight the cost and margin levels. Further, in the second step, it is necessary to link activities and products: for each activity, a cost driver will be selected and monitored (for example, the number of orders, and reference quantities). This driver will be the unit used to allocate the total cost of the activity. Some drivers will not be used to avoid heavy models. A typical driver is preferred for each activity. For each studied activity, the model will, therefore, specify the consumed drivers. The drivers are indicators that make it possible to highlight the cost and margin levels. The activity driver is an indicator that measures the resources consumed by the company’s activity and identifies the cost/revenue ratio. The cost driver is an indicator that determines the level of costs through the organization of the activity.

The ABC method can be summarized in two meta-drivers: the activity driver, which measures the resources consumed by the company’s activity and identifies the cost/product follow-up ratio, and the cost driver, which determines the level of costs through the organization of the activity. Through the analysis of these drivers, the ABC method deconstructs the different types of activities by allocating costs to products more efficiently. By knowing these indicators, a manager can make strategic decisions: regarding cost reduction, reorganization, etc. It is this high granularity, in the knowledge of processes and drivers, that enables the identification of sobriety paths. We use the ABC analytics frame to decompose the problem of sobriety characterization.

Consequently, we can apply this specific method to make precise the features of the life cycle assessment of the services/products.

In this method, which we will describe in detail below, each productive process (called inductor *I*) is split into activities (*a*), which are themselves split into tasks (*t*) involving the use of resources (*r*) corresponding to costs (*c*). The activities are, therefore, a function of the tasks and resources consumed, ultimately inducing a cost, i.e., the existence of a composite function *I*:

I=f(a)witha=g(t,r)=f(g(t,r)) (1)

The production (P) of a given good or service, therefore, corresponds to the sum of the n inductors (I), necessary for the production of said good/service. It also becomes possible to consider each driver in a differentiated way.


$$P=\sum_{i=1}^{n}I‍$$ (2)

Besides this, there is the question of the production purpose. Seeking an improvement in a production process makes sense only if it is justified. No one produces if there is no interest in producing a potential for satisfaction. In other words, it would then be a question of considering production as necessary to respond to an expectation [46] and to satisfy an expectation [47], in an entirely Maslovian logic. In this context, satisfaction is therefore a function of production. So, if *S* = *z*(*P*),

$$S=f(\sum_{i=1}^{n}f(a)\circ g(t,r)‍)$$ (3)

We, therefore, find in the satisfaction function, a reference to resources and their use as a composite function of activities. Each productive process must, therefore, correspond to a satisfaction function (S). Similarly, each satisfaction function encounters a corresponding production function (P) at a given interval.

We consider that a satisfaction function is verified, to proceed from one stage to another. Hence, the next production cycle will be engaged if the satisfaction function of the previous level is verified.

**Fig 2:**
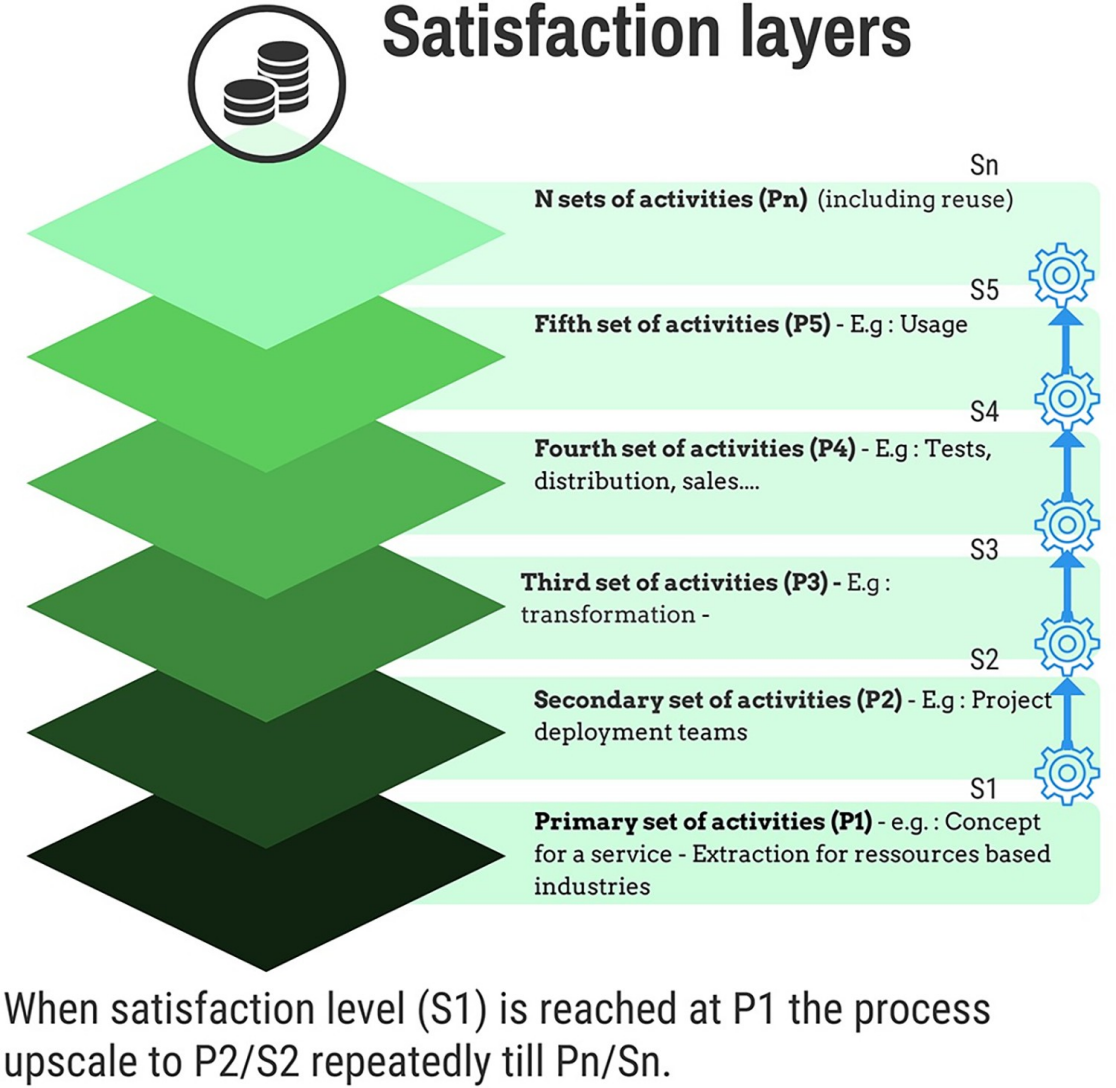
Principle of satisfaction layers.

For example, in artificial intelligence, we can start to perform a calculation if we already have a technical and human ecosystem to do so. We need to account for these interactions, these sets of successive phases, by expressing the stacking of satisfactions, i.e., at which point in the activity cycle the change in the level of satisfaction occurs, i.e., the engagement of new activities integrating the first activities, but going beyond, happens. Therefore, each finished product/service comprises n levels of satisfaction as a function of the complexity of the productive processes involved, knowing, as we have said, that each of these stages corresponds to activities, which are linked to the use of resources and costs. Yet, we know that there is a potential for sobriety at each iteration (Fig 2).

This gives a good idea of the exponential depth of the potential sobriety wells that apply in this way to a given end-user and to the entire chain of different users involved in producing a good or service. In other words, there is a potential for sobriety at each stage of the production cycle of a product/service: from concept to marketing. Therefore, it is necessary to consider the diversity of actions that ultimately compose each product and service. For this purpose, we have adapted the well-tried product/service life cycle method.

### Life cycle analysis

Life cycle analysis (LCA) is the most advanced tool for global and multi-criteria assessment of environmental impacts. This standardized method enables the measurement of the quantifiable effects of products or services on the environment. LCA identifies and quantifies, throughout the life of products, several parameters, named as drivers. It assesses the potential impacts and then interprets the results obtained, according to its initial objectives. Its robustness is based on a dual approach: whether it is a good, a service, or even a process, all the stages of the life cycle of a product are taken into account for the inventory of flows.

An LCA is analyzed, based on several criteria, for analyzing incoming and outgoing pertinent parameters of the system. We every parameter call that contributes to the manufacture of the product and everything that comes out in terms of pollution as “drivers”.

**Fig 3:**
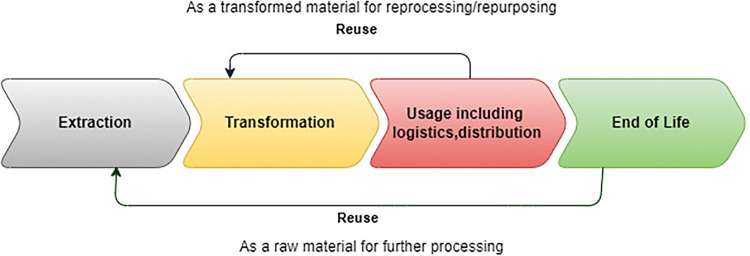
Production cycle: Extraction → Transformation → Use (distribution—provision) → Re-use → End of Life (Destruction).

Collecting flow information is an important step in LCA. They are quantified at each stage of the cycle, and they correspond to indicators of the potential impact on the environment. The complexity of the phenomena involved, and their interactions are a source of uncertainty about the real value of the impacts, which is why they are called potential. Each component of the cycle is seen as a subsystem, whose satisfaction function can be mathematically defined. This function contributes as a step validation criterion for each part of the cycle. As we said, the ABC method implies the existence of drivers corresponding to the splitting of each life cycle (c) of a production operation (whether it is for goods or services) into activities, themselves corresponding to the mobilization of resources, at a cost that is both economic (the price of mobilizing these resources) and operational (the volume of use of these resources). The production life cycle (or product life cycle) is standardized [48] and shared by all economic actors, which is usually divided today into four main phases (this can be more, but the addition of a phase does not particularly change the nature of the present analysis). This is of course, found at each stage of the production cycle, i.e., the stages that have all the elements to ultimately lead to the production (P) and provision of the product/service. The production cycle is divided into five iterative phases (Fig 3): Extraction → Transformation → Use (distribution, provision) → Re-use → End of Life (Destruction).

Logically, at each stage of the process, an optimal level of satisfaction for the end user concerned must be achieved, at a given level of the life cycle of the product, to move on to the next stage. In short, it is a matter of reasoned decision-making linked to the stage of the production cycle where each person is [49]. For example, a product extracted from given robustness standards will be expected to be capable of entering a given production process [50]. Further, and in terms of meeting an expectation that is found at each stage of the production process, it can be said that sobriety (S) is a function of user satisfaction, found at each stage of the production cycle and life of the product and is the marker of the passage to the next stage of the cycle. In other words, as soon as the level of satisfaction (volume, robustness, etc.) is reached, it becomes possible to move on to the next step [51]. We logically deduce that there are entry markers of the satisfaction function, which designate an operational floor (saddle point). In the logic of transition to an industrial operationality, it will be necessary for each process to identify these points, or markers. For our part, we hypothesize that there are untapped deposits of sobriety in all current economic processes, deposits that we have identified and modeled for AI, to demonstrate our concept within the framework of the present paper.

For each stage of the product life cycle, there is thus an end user who will consume/use each driver and its components (the resources), to move to the next stage. We, thus, postulate that reuse is part of a Schumpeterian logic of creative destruction [52]. We assume that there is, for each stage (E) of the product life cycle (c), a set of variables, x and y, corresponding to the drivers mentioned above. We distinguish, for each operation, permanent drivers (x), which are found in all operations, whatever the stage of the life cycle, and impermanent drivers (y), which are found only at certain stages. We will see below the application of this principle, in the case of artificial intelligence. Each driver d is, therefore, a function of x and y. Similarly, each driver involves costs. We set d = f (m, v). Each stage of the life cycle has a logical link. It is, therefore, necessary to satisfy the extraction to move on to the transformation phase. For example, like the Maslovian approach, having enough material to transform [53] each step involves the satisfaction of a given need to move on to the next one. In this context, we call satisfaction S (x, y) as any function S, corresponding to the expectation of a given end user at a given time in the cycle. We postulate that there exists in this context a floor (minima) of satisfaction that makes it possible to pass to the next stage of the life cycle without this floor requiring the total consumption (maximum) of the resources as implied by each driver. It is therefore possible to sufficiently satisfy a user to ensure the continuity of the cycle, without consuming all the available resources for each driver, which we call a source of sobriety. We, therefore, seek for each satisfaction floor a resource consumption ceiling for which any higher consumption will not bring an increase in the level of satisfaction.

To illustrate and demonstrate our point, we have widely applied ABC analytics to the industrial process of artificial intelligence. From the viewpoint of the granularity of our demonstration, we are at the “meso” level, without ignoring the complexity of the anatomy of AI systems [54].

We are looking for optimal control of the industrial process, favorable for the economy (i.e., economical sobriety), through the search for technical and energetic sobriety characterization. That’s why we convert the economical view (i.e., ABC analytics model) into a mathematical model, formulated as an optimal control for the AI process.

### Mathematical model

We model the considered process with several variables and functions:

1. data, named *d*
2. AI algorithm, named *a*_*AI*_
3. hardware (having a depreciation component *m*_1_ and maintenance *m*_2_) with an evolutive index named: *i*(*m*_1_, *m*_2_)
4. a given energy consumption, *e*
5. human inputs, *i*_*h*_.

We can mathematically define the concept of *satisfaction*, applied for both each part of the process and for a given end-user. We assume that end-user satisfaction depends on the combination of these elements, at given times in the product life cycle. We define the satisfaction function, as a mathematical function depending on *x*, the permanent drivers, and *y*, the impermanent drivers. Hence, with: *x* = *f*(*e*, *i*_*h*_) and *y* = *g*(*d*, *a*, *h*), knowing *h* = *i*(*m*_1_, *m*_2_), any function *S* corresponding to the expectation of a given end-user at a given moment of the cycle, is:

S=S(x,y) (4)

We also define the truth matrix, named *P*−*matrix* (Table 1). It allows us to locate the presence of predictors by “moment” of the cycle. Precisely, it connects the several processes of the cycle with the drivers. We can locate the presence of predictors for different moments of the cycle.

**Table 1:** The P-Matrix (Predictors per cycle “moments”).

	Moment 1	Moment 2	Moment 3	Moment 4	Moment 5
**Cycles/Frames predictors**	d	a	*h* = *i*(*m*_1_, *m*_2_)	e	*i*_*h*_
**Extraction**	X	Maybe		X	X
**Transformation**	X	X		X	X
**Exchange**	X	X	X	X	X
**Reuse**		X	X	X	X

Obviously, by modeling the problem as a dynamic system, we set two kinds of variables continuous time and discrete event systems, depending on their nature. “Discrete variable” means variables taking their values in a discrete set and “continuous variable” means variables taking their values in a continuous set. The notion of hybrid dynamic systems appears where the necessity to develop a theory of melting continuous and discrete signals is stressed. When a discrete event occurs, it may change the continuous dynamic and thus discontinuities may appear in the vector field and/or in the continuous state.

**Fig 4:**
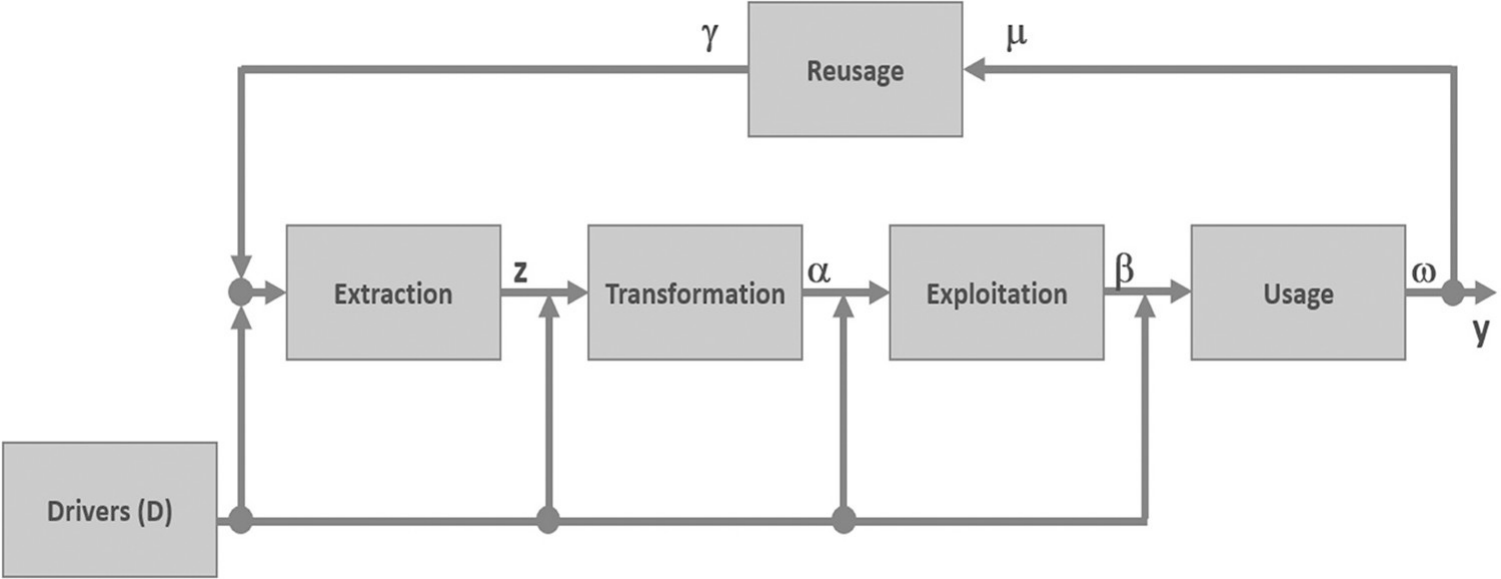
Process life cycle.

Fig 4 describes the process life cycle considered in this work. It is formed by processes such as extraction, transformation, exploitation, and usage. Each of these processes takes as input some drivers (such as datasets, algorithms, computational resources, and human resources) and the outputs of each process. The problem that this process life cycle resolves is expressed in Eq (5).

$$\begin{array}{c} \underset{\Theta }{max}\;S\;s.t.\;z=Extract(D,\gamma ),\\ \alpha =Transform(D,z),\\ \beta =Exploit(D,\alpha ),\\ \omega =Use(D,\beta ),\\ \mu =\omega \backslash y,\;\gamma =Reuse(D,\mu ),\\ S=(S_{y},S_{\omega },S_{\beta },S_{\alpha },S_{z},S_{\gamma }),\\ \Theta =(\theta_{\omega },\theta_{\beta },\theta_{\alpha },\theta_{z},\theta_{\gamma }) \end{array}$$ (5)

where *S* is a multi-objective *satisfaction* function (*S*_*y*_, *S*_*ω*_, *S*_*β*_, *S*_*α*_, *S*_*z*_, *S*_*γ*_), with each objective function corresponding to the satisfaction of each process. *Θ* = (*θ*_*ω*_, *θ*_*β*_, *θ*_*α*_, *θ*_*z*_, *θ*_*γ*_) are the parameters encompassing each process model. (*z*, *α*, *β*, *ω*, *μ*) are the outputs of the processes of extraction, transformation, exploitation, usage, and reuse, respectively. The output of the process life cycle is *y* and is *ω*\*μ*. In this work, we limited ourselves to the transformation process. It is pertinent to highlight whether sobriety exists at this stage, as this phase is costly. We define Sobriety as the energetic, digital, economic reduction of costs without any change or few changes in satisfaction. Then, we want to quantitatively characterize the *sobriety deposit* of the transformation step inside the LCA. The next subsection is dedicated to defining the appropriate method.

## Methodology for green AI procedure: Sobriety deposit characterization

**Fig 5:**
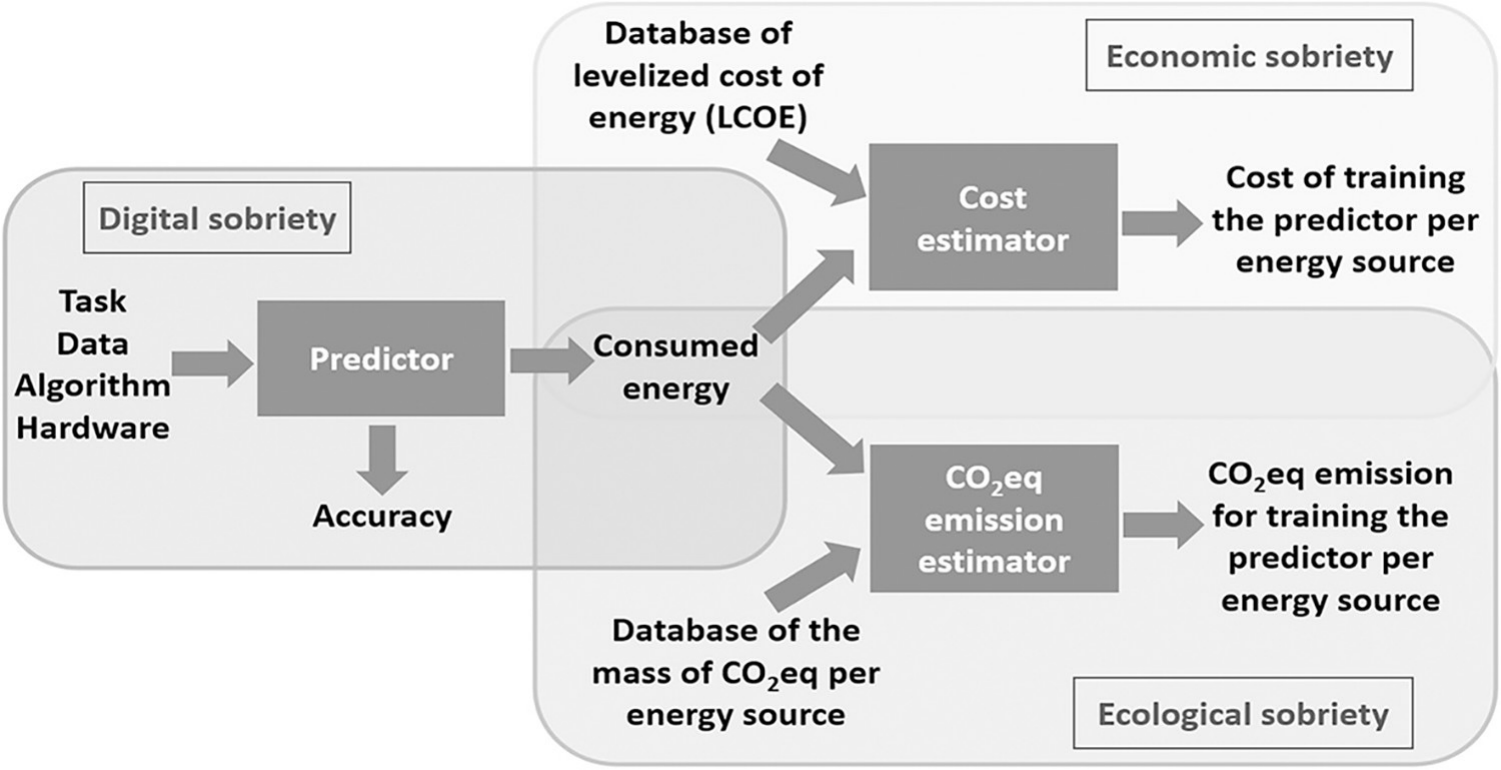
Flow chart for digital, economic, and ecological sobrieties.

In this section, we explain the specific method used for mathematically identifying and characterizing the satisfaction function of an AI process. As the function depends on several variables and the optimization is based on a multi-objective method, we focus only on the *data reduction* aspect. As a proof of concept, we would like to highlight how reducing the input data required to train an ML model impacts the reduction of the cost and the *CO*_2_*eq* emission. For this purpose, we follow the logic flows indicated in Fig 5.

For this purpose, we first consider the CIFAR-10 dataset [21]. The CIFAR-10 dataset consists of 60,000 images of 32×32 pixels, representing 10 classes. Each class has 6,000 images. Out of these 60,000 images, 50,000 are used for training purposes, and the remaining 10,000 are used for testing purposes.

To show the effect of digital sobriety, out of the 50,000 images available in the CIFAR-10 dataset for training a classifying model (which is described in the sequel), some subsets of these images are fed to the classifying model to show the performance of this classifier and the energy consumed as a function of the input data size.

Concretely, (4,992; 12,480; 24,960; 37,440; 44,992; 49,984) images are employed and they roughly represent (10%, 25%, 50%, 75%, 90%, *and* 100%) of the training dataset.

**Fig 6:**
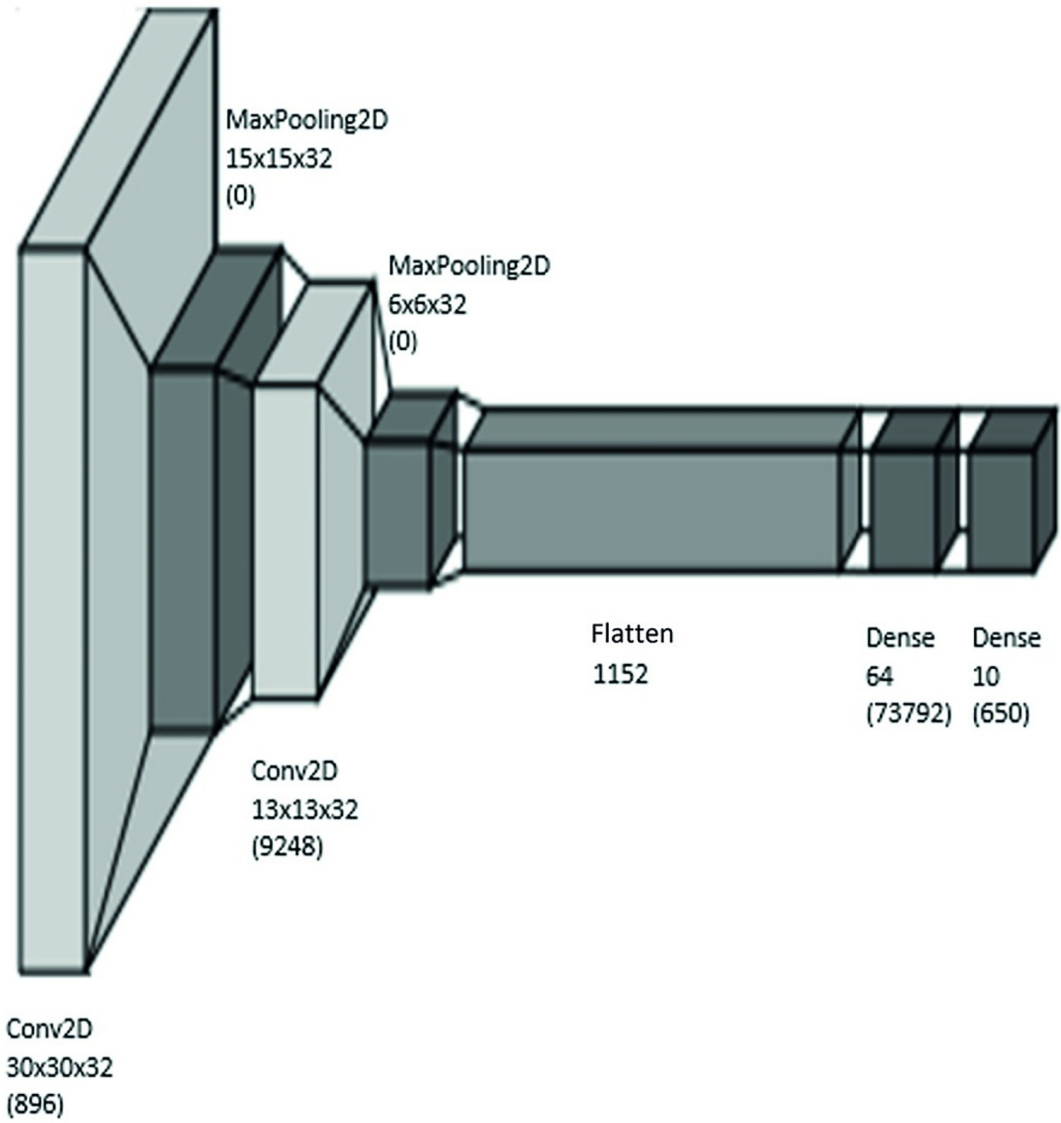
CNN architecture.

In this work, we classify the images of the CIFAR-10, using a CNN-based classifier model, which is implemented using Python 3.8.10 and the following libraries: TensorFlow 2.9.1, Keras 2.9.0, Scikit-learn 1.1.2, NumPy 1.23.1, and MatPlotLib 3.5.3. The classification model is presented in Fig 6. This model first receives images of 32×32 pixels, with 3 colors from the CIFAR-10. These images pass through a 2D-convolutional layer, with a filter size of 3×3 and 3 channels. This is followed by a 2D-max pooling layer of size 2. Its outputs now go through another 2D-convolutional layer of size 3×3 with 3 channels. Thereafter, its outputs are max pooled with size 2. Now, the outputs are flattened to go through a fully-connected layer with 64 neurons. Finally, it is followed by another fully connected layer with 10 neurons (since the total number of classes is 10). The loss function to be optimized is the cross-entropy between the actual labels and the predictions. The employed batch size is 64 (images), and each experience consists of about 900 epochs.

The training performance is measured by accuracy, which is the ratio of the successful predictions over all the realized predictions. That is,

$$accuracy=\frac{Truepositive+Truenegative}{Truepositive+Truenegative+Falsepositive+Falsenegative}$$ (6)

where *true positive*, *true negative*, *false positive*, and *false negative* signifies correct positive-value estimation, correct negative-value estimation, incorrect positive-value estimation, and incorrect negative-value estimation, respectively.

The hardware employed to train the algorithm is an HP Z240 SFF. It has a CPU of Quad-core Intel i7-7700 3.60GHz with 2 threads per core. The memory size is 16GB, and the storage devices consist of one 500GB-SSD and one 1TB-HDD. The CNN algorithm is run over Ubuntu 18.04 (the operating system).

As explained earlier, different sizes of training datasets are employed to observe whether there is a data size from which the model performance does not significantly change, but the energy consumed increases significantly. The energy consumed depends on the data employed, the ML algorithm, the hardware specification, as well as the model-training duration. In this way, we can find the desired size of the training dataset.

**Fig 7:**
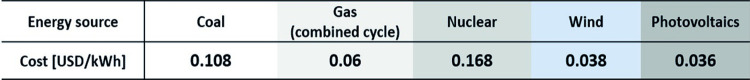
Conversion tables from energy to cost [55] and from energy to *CO*_2_*eq* emission [56].

Now, using the *levelized cost of electricity (LCOE)*, published by Lazard [57], we can estimate the cost associated with the energy consumed that corresponds to each size of the training dataset (see Fig 7).

**Fig 8:**
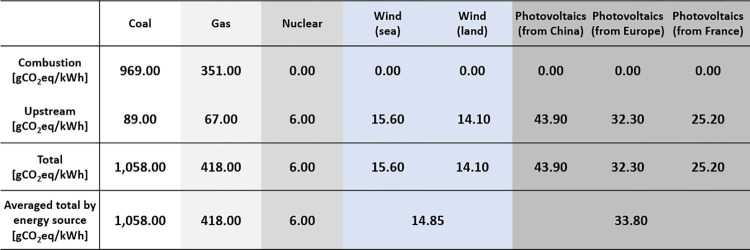
Conversion table from energy to CO2eq emission.

Further, we use a database published by ADEME (Agence de la Transition Ecologique) [56], the French Agency for Ecological Transition, to estimate the mass of *CO*_2_*eq* emitted by each major energy source (see Fig 8).

## Results and discussion

In this section, we illustrate the results obtained from the methodology explained in the last subsection of the Section “Frame and Methods”. Such results are shown in this section: digital sobriety, economic sobriety, and ecological sobriety. For each sobriety class, we show and discuss the results.

We define each component of sobriety. As we want to create a paradigm where technological-economic efficiency and environmentally inspired sufficiency can be reconciled, we give intercorrelated definitions in the sufficiency triangle.

**Fig 9:**
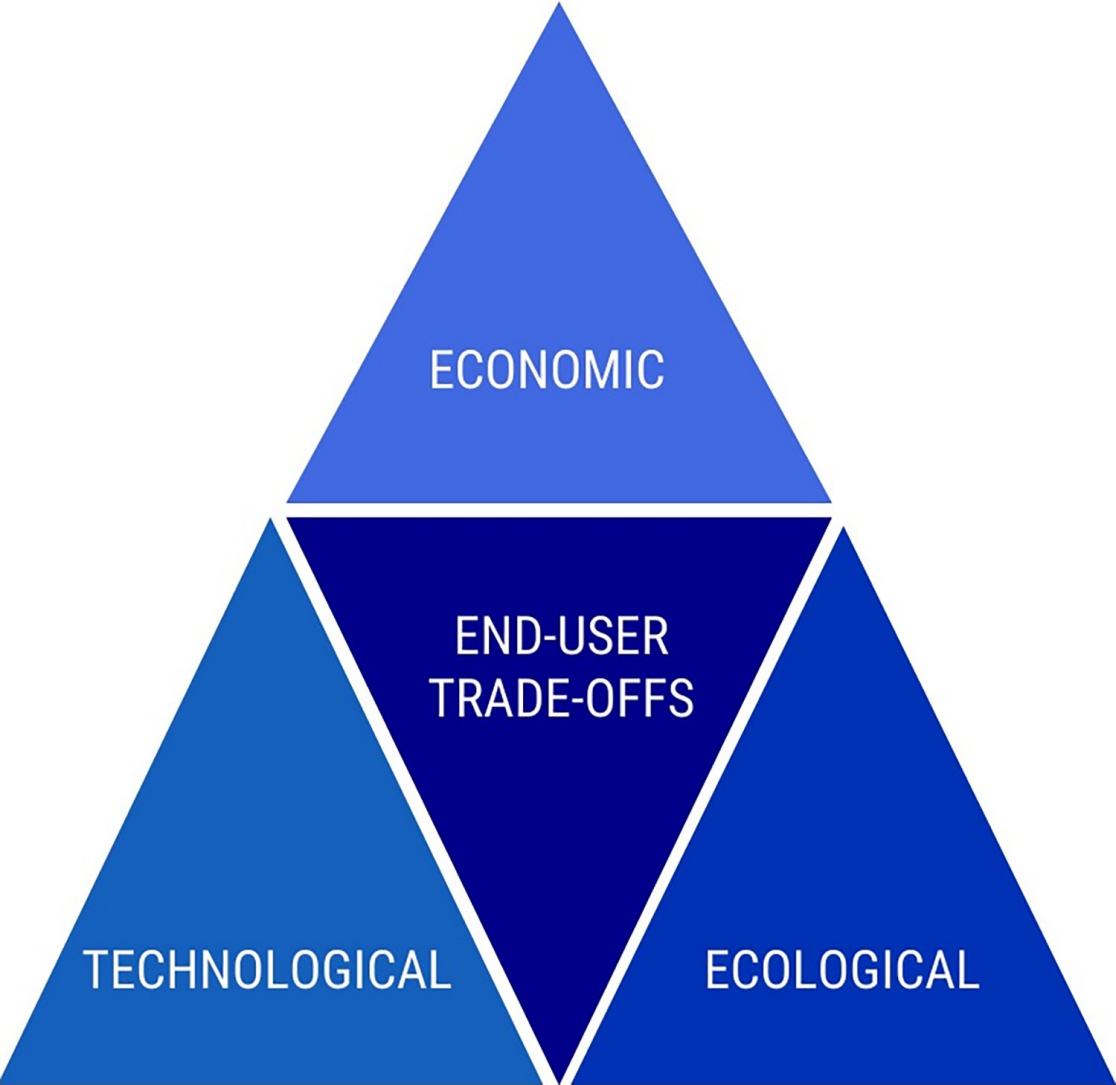
Moderation triangle through sobriety.

We extend the concept of sufficiency from Thomas Princen [58]. Technological (numerical in this study) sufficiency can be defined as a significant reduction of the resources’ costs without any quantitative difference in the satisfaction indicators. Here, accuracy will be used as a satisfaction indicator, as stated in section 2. From this technological sufficiency, we define economical sufficiency as the reduction of a cost and/or margin enhancement, for the concerned activity. At the same time, ecological sufficiency can be defined as quantitative ecological footprint reduction. It is measured here as the total greenhouse gas (GHG) created from activities and tasks, expressed as carbon dioxide equivalent CO2eq. That’s why we define a frame called the Moderation triangle through Sobriety (Fig 9).

### Digital sobriety

**Fig 10:**
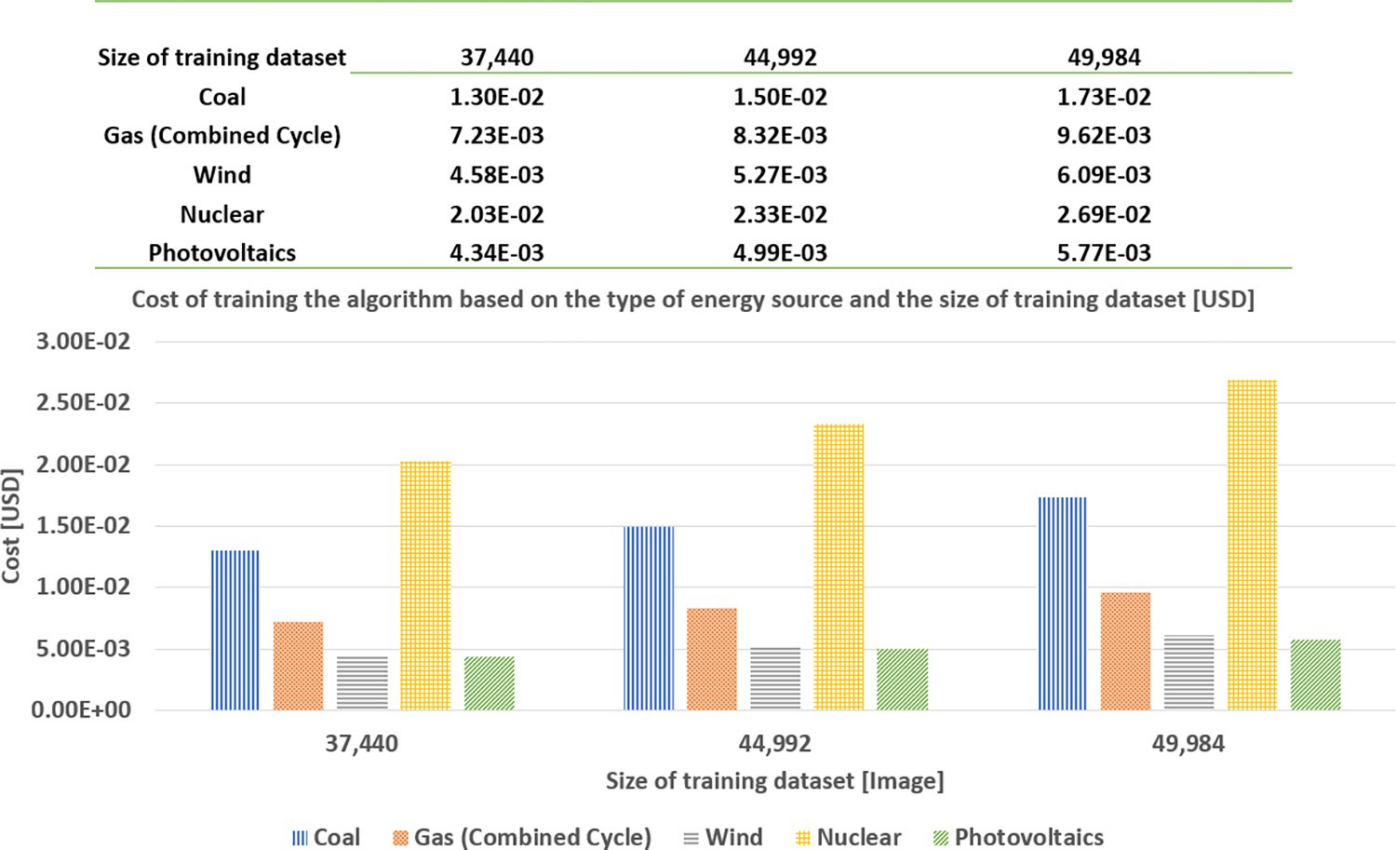
The cost of training the algorithm is based on the type of energy source and the size of the training dataset.

As indicated in Fig 5, in this section, we show the results obtained from the classification predictor, in terms of accuracy and the consumed energy from the tuple of (task, data, algorithm, and hardware). Effectively, in Fig 10, we compare the accuracy and the energy consumption associated with training the CNN algorithm, depending on the size of the dataset employed. The values on the horizontal axis are (4, 992; 12, 480; 24, 960; 37, 440; 44, 992; 49, 984), which approximately represent (10%, 25%, 50%, 75%, 90%, and100%) of the training dataset from the CIFAR-10 [21].

**Fig 11:**
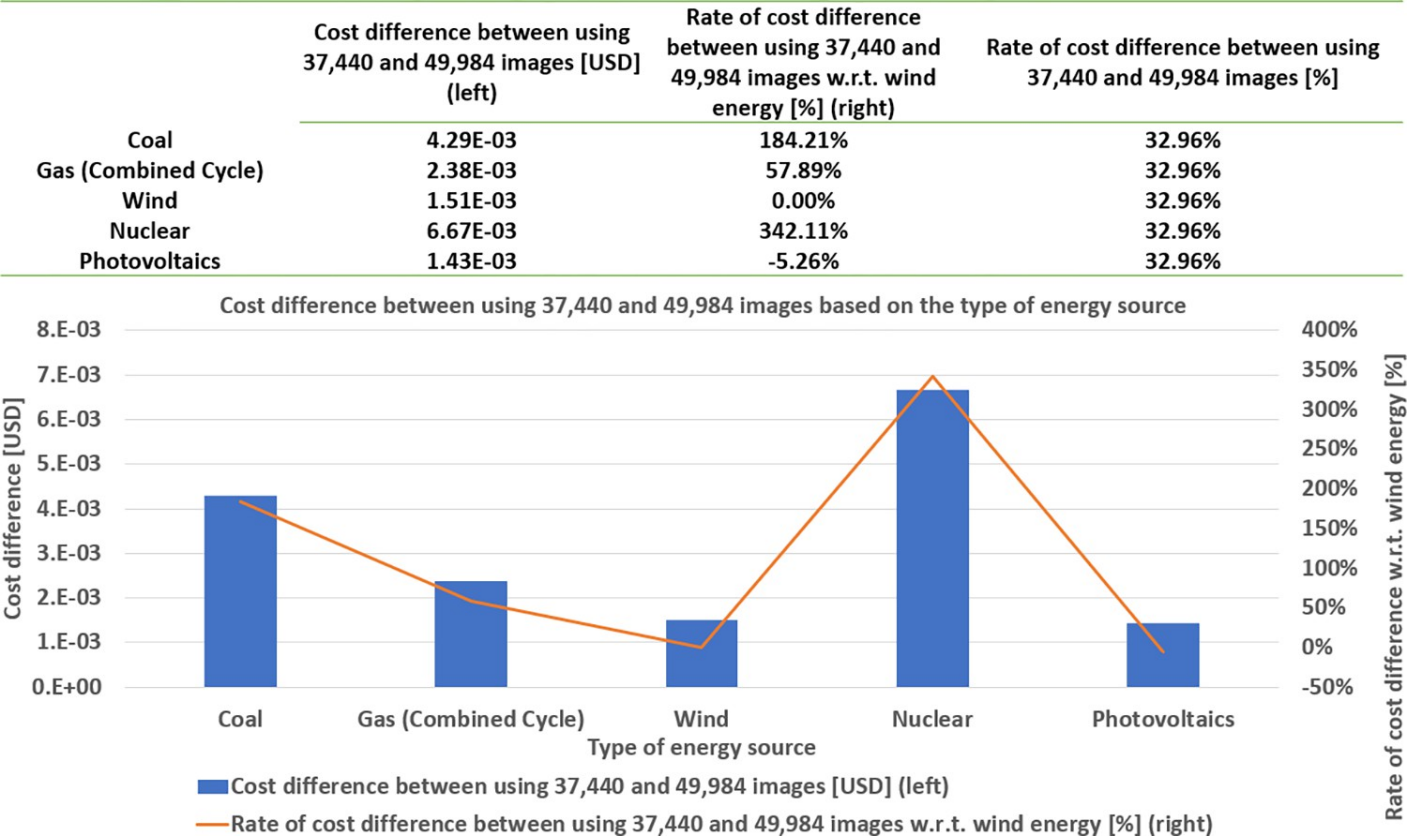
Cost difference and its rate between 37,440 and 49,984 images based on the type of energy source.

The left vertical axis illustrates the energy consumption in kWh. The right vertical axis shows the accuracy in %, corresponding to the considered sizes of the training dataset. All the values shown in Fig 11 are the average values of running 10 experiments for each dataset size.

We first observe that the accuracy monotonically increases as the size of the training dataset increases. That is, by using (4, 992; 12, 480; 24, 960; 37, 440; 44, 992; 49, 984) images to train the model, it is possible to respectively achieve (53.77%; 59.19%; 63.57%; 66.24%; 66.99%; 67.86%) of accuracy, requiring (0.02; 0.04; 0.08; 0.12; 0.14; 0.16), respectively. Hence, we perceive that, from about 75% of the total size (i.e., 37,440 images), the accuracy curve reaches a plateau between 66% and 68%, not showing a significant improvement, even if we feed more data to train the model. We observe that this is not the case with energy consumption. It monotonically increases with no sign of a plateau.

**Fig 12:**
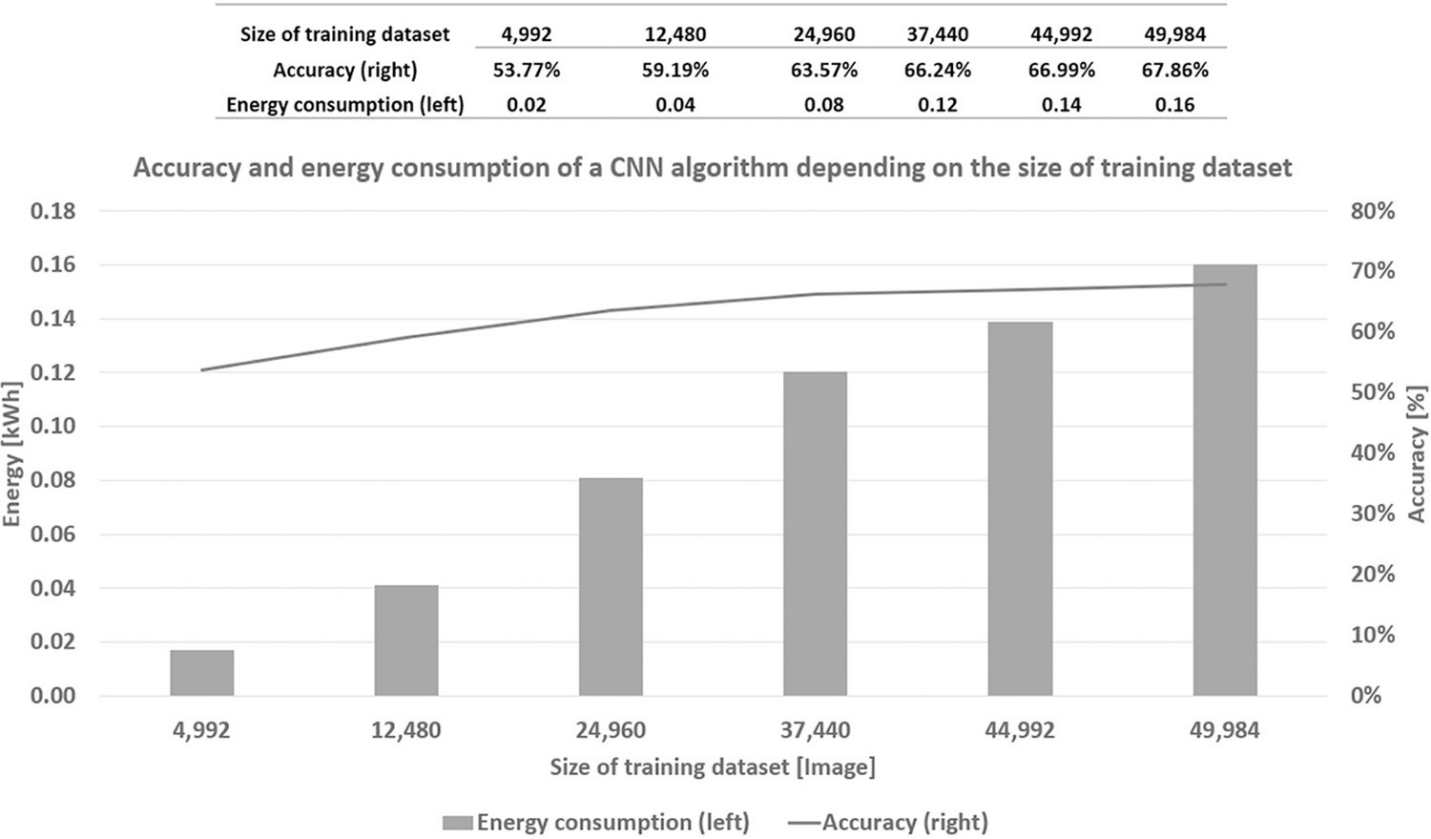
Digital sobriety.

Therefore, adding more data to train the model does not significantly improve the accuracy, while always requiring more energy consumption. Thanks to this comparison between the accuracy and energy consumption, we can seek digital sobriety (Fig 12), in the sense of establishing a reference to reduce the amount of data needed to train the model, while guaranteeing a satisfactory performance.

### Economic sobriety

Recall from Fig 5 that we can estimate the cost of training the model from both the energy consumed (for each dataset size) and the database of levelized cost of energy (LCOE) as a reference, published in [57] and shown in Fig 8, to seek economic sobriety. We have seen in the sobriety deposit characterization section that the model performance (in terms of accuracy) reaches a plateau. In this section, we concentrate on this plateau to analyze the cost reduction while satisfying the algorithm performance. Therefore, in Fig 11, the horizontal axis shows three sizes of the training dataset (corresponding to the accuracy-plateau domain). For each size of the training dataset, we consider five different energy sources: coal, gas (combined cycle), wind, nuclear, and photovoltaics.

On the other hand, the vertical axis shows the cost of training the model, based on the type of energy source and the size of the training dataset, in US dollars. From Fig 11, in general, we observe that the cost of training the model increases with the increase of the dataset size regardless of the type of energy source (as this was the case with the energy consumption). Further, we notice that the cost is sorted in descending order by the type of energy source as nuclear, coal, gas, wind, and photovoltaics. Next, Fig 14 shows two types of results: the cost difference between using 37,440 and 49,984 images for training the model, and the rate of the cost difference between using 37,440 and 49,984 images concerning wind energy. The values of 37,440 and 49,984 images correspond to the sizes of the training dataset for the beginning and end of the plateau in the accuracy curve, as shown in Fig 11. As was the case with the cost of training the model, for the three sizes of training dataset, corresponding to the plateau, the difference of this cost is again ranked in descending order as nuclear, coal, gas, wind, and photovoltaics (see the values of the left vertical axis). Moreover, we compute the rate of the cost difference between using 37,440 and 49,984 images concerning wind energy. The cost of wind energy is taken as the reference value (instead of the photovoltaic energy, even if the latter one is smaller) because we use wind energy as the reference for the study of ecological sobriety, which is shown in the next section. The rates of cost difference are ordered in a descending manner, as in the cases of the cost of training the model and its differences (see the right vertical axis). The rate of the cost difference of nuclear energy is about thrice higher than that of wind energy, whereas the cost difference of coal is about twice higher than that of wind energy.

In addition, we notice that we can approximately achieve 33% of economic sobriety while keeping nearly the same model performance (in terms of accuracy).

### Ecological sobriety

As shown in Fig 6, we can estimate the mass of *CO*_2_*eq* emission for training the model from both the energy consumed (for each dataset size) and the database of the conversion from the consumed energy to the mass of *CO*_2_*eq*, published by ADEME [56] and shown in Fig 12, for seeking *ecological sobriety*. As previously done, for both digital and economic sobrieties, we concentrate our analysis only on the data sizes corresponding to the accuracy plateau, as shown in Fig 11 (i.e., (37,440; 44,992, 49,984) images).

**Fig 13:**
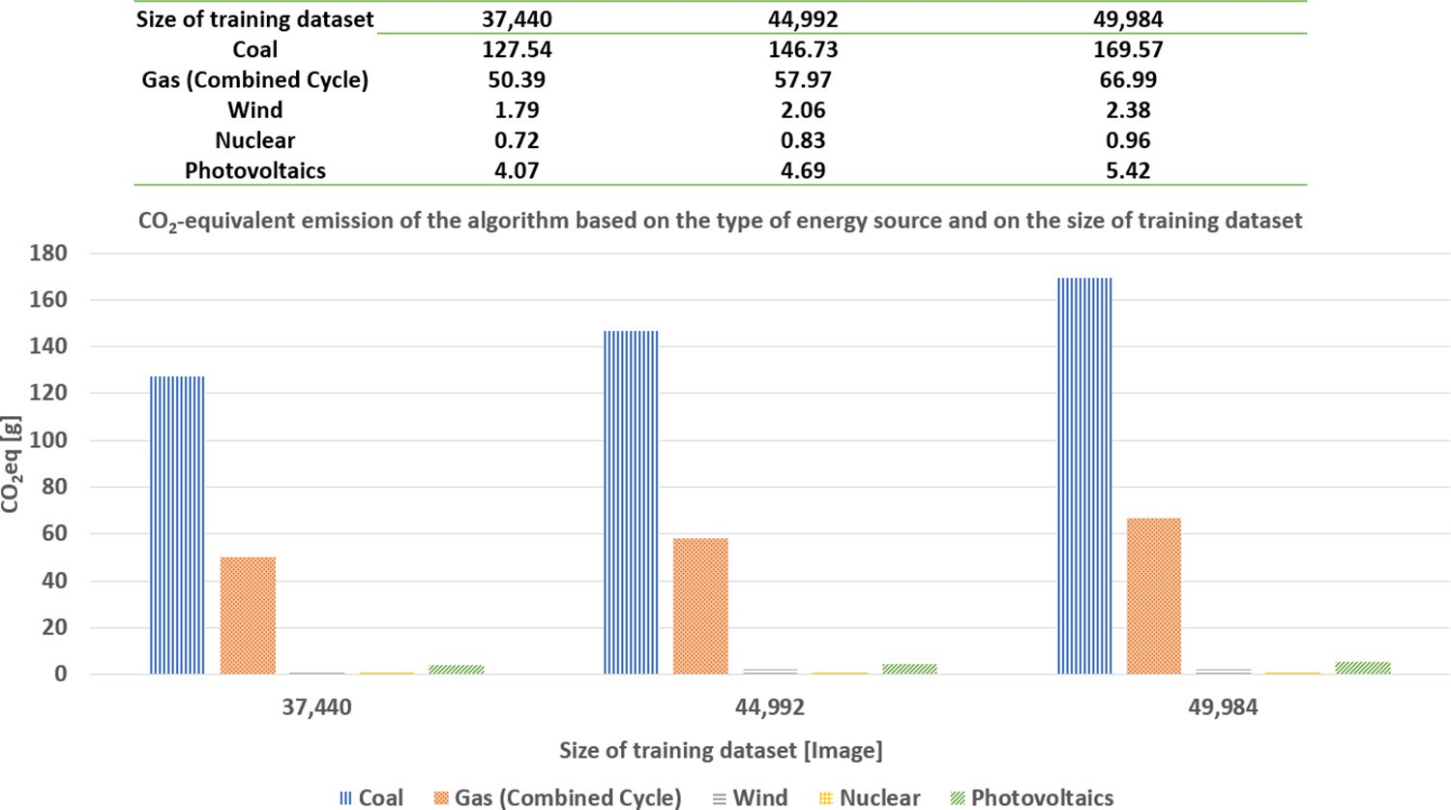
CO2eq emission for training the algorithm based on the type of energy source and on the size of the dataset.

**Fig 14:**
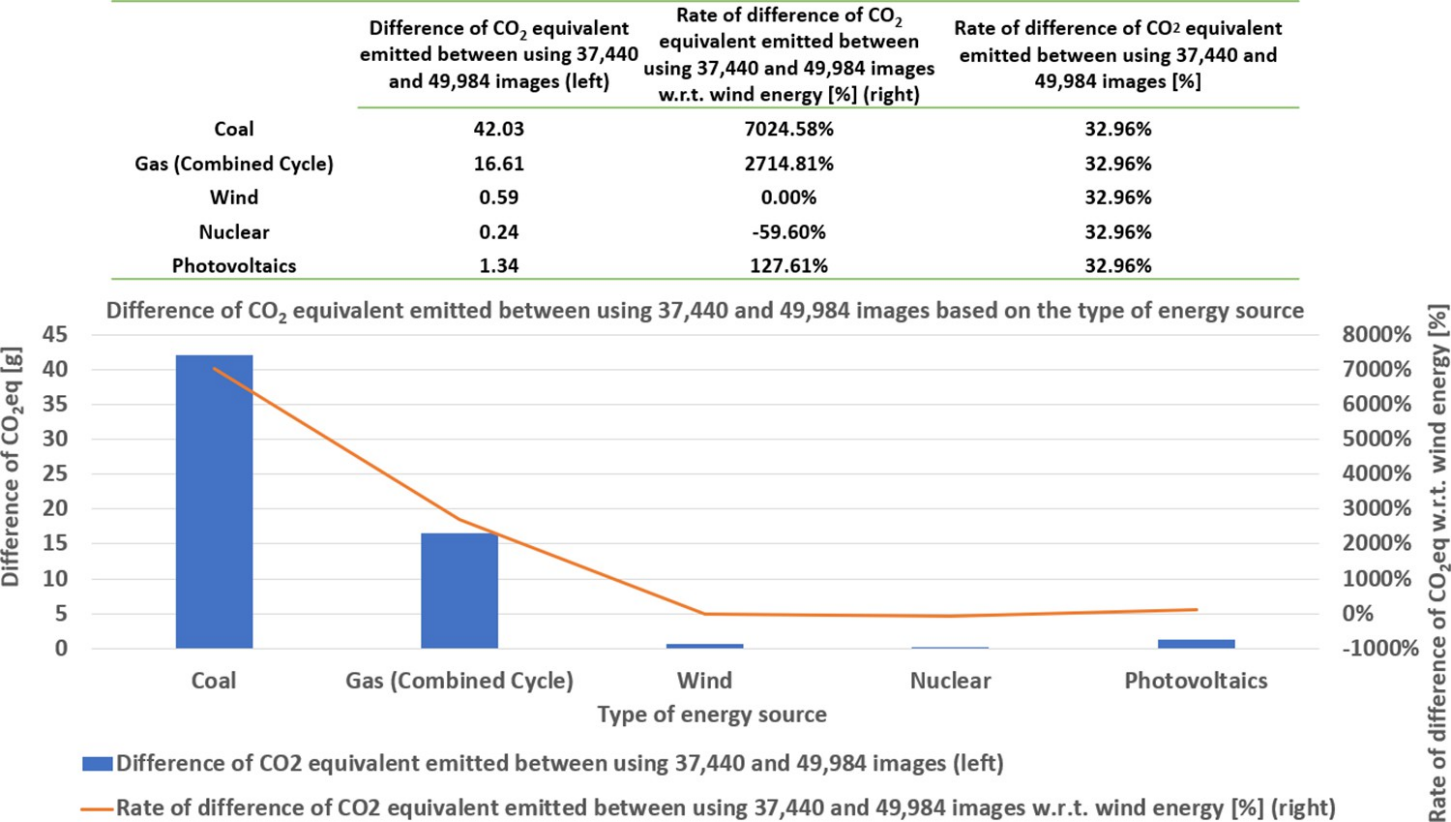
Difference of CO2eq emission and its rate between 37,440 and 49,984 images, for training the algorithm, for various types of energy sources.

In Fig 14, we show the CO2eq emission of the algorithm, based on the type of energy source and the size of the training dataset. This emission is measured as a mass in grams. First, the values of the horizontal axis correspond to five types of energy sources for three dataset sizes (corresponding to the domain of the accuracy plateau, as shown in Fig 11). In general, we observe that, as the dataset size increases, the CO2eq emission also increases, regardless of the type of energy source (as this was the case with the consumed energy (see Fig 11) and its associated cost (see Fig 13)).

Further, the mass of emitted CO2eq is sorted in descending order, according to the type of energy source as coal, gas, photovoltaics, wind, and nuclear. Fig 14 shows two types of results: the mass difference of emitted CO2eq, emitted between using 37,440 and 49,984 images, based on the type of energy source, and the rate of mass difference of CO2eq emitted, concerning wind energy. First, the mass difference of emitted CO2eq, between using 37,440 and 49,984 images, is ranked in descending order as coal, gas, photovoltaics, wind, and nuclear (see the values of the left vertical axis). Moreover, we compute the rate of the mass difference, between 37,440 and 49,984 images, concerning wind energy. The mass of CO2eq emitted, corresponding to wind energy, is considered as the reference value (instead of the nuclear energy; even the latter one is smaller) because, even though nuclear energy emits the smallest amount of CO2eq, this does not necessarily imply that it is the least polluting energy (for this reason, we should use a metric other than the mass of emitted CO2eq). This is a perspective for future work. Therefore, we take wind energy as the reference energy source. Now, we observe that coal roughly emits seventy times more CO2eq than wind energy, whereas gas roughly emits twenty-seven times more CO2eq than wind energy. Again, we observe that we can approximately achieve 33% of ecological sobriety while retaining nearly the same model of performance (in terms of accuracy).

## Conclusion and future work

To increase efficiency, it has been postulated by numerous studies that artificial intelligence would require more and more resources [59]. We have shown in this paper that this is not the case and that it is indeed possible to preserve the efficiency of AI processes while drastically minimizing the need for resources. In particular, we have demonstrated that it is possible to be digitally sober, by controlling the size of the training dataset, while guaranteeing nearly the same model performance. As subsequent results, and as a consequence of having searched the digital sobriety, we can be both economically and ecologically sober. This argument is evidenced by optimizing a CNN-based classification model, with various sizes of training datasets, and subsequently collecting the corresponding accuracy and energy consumed values. The levelized costs of energy (LCOE) and the mass of emitted CO2eq, published by Lazard [55] and ADEME [56], respectively, are employed to estimate both the actual cost and the actual mass of emitted CO2eq for different types of energy sources.

Through this work, we first show that wind energy is three times less costly than nuclear energy and twice less costly than coal energy. We further report that wind energy emits seventy times less CO2eq than coal energy and twenty-seven times less CO2eq than gas energy. We further show that we can achieve nearly 33% of digital-economic-ecological sobrieties by reducing the dataset for training the model to predict image classification.

We consider that this preliminary work sets the stage for many improvements. For example, we intend to consider dynamic prices, instead of static prices used here (in the form of LCOE), to measure a more realistic economic impact. Indeed, one of the natural perspectives of this work, carried out in static cost, is to extend it to the dynamic test case, by integrating the effects of the energy market as an agent external to the life cycle.

Concerning the different drivers that impact the Satisfaction function, it is necessary to be able to further explore each of their contributions, in particular, those associated with energy aspects. If we place ourselves in a logic of reducing the production of GHGs during an AI process, we will have to consider the metrics, more subtle than those frequently used. Indeed, we have used the CO2eq emission to seek ecological sobriety. However, we consider that this measure does not fully render the actual ecological impact as, for instance, nuclear energy causes least pollution in terms of CO2eq, but can generate other adverse pollution effects, through radioactivity.

Hence, indicators that consider not only emissions but also “eco-systemic” risk potentials are yet to be designed. Moreover, in the present work, we considered one type of deep learning-based classification model. However, in future operations, we would like to observe more complex classification models such as AlexNet [60] or ResNet [61]. The attainment of having decomposed the life cycle of an AI process into different tasks means that we can finely characterize the notion of overall satisfaction. It characterizes the satisfaction linked to each task by relevant criteria. It would also be interesting to extend the definition of the phenomenon to a more global conceptualization. Even though limited to the “data” variable, this study has proven the existence of a virtuous circle, whose governance is driven by digital sobriety, called the “sobriety triangle”.

Further, knowledge of the full cost of AI utilization, in the business environment, guarantees the adherence of economic agents to more frugal practices, and more respect for the environment; if they can price better, they will be able to invoice and optimize better. Economic agents expect an approach that (a) makes the costs involved and (b) makes the impact of optimizing these costs on margins, both visible in the balance sheets. They also require (c) the certainty that these improvements can be efficiently deployed i.e., the envisaged processes generate operational improvements while preserving the formers’ efficiency and excellence, and thus, they can be translated into industrial processes. A full-cost model can “trigger” the use of the right resources at the right moment, while delivering adequate results. Similarly, it may allow decision-makers to choose simpler solutions instead of complex ones, that, nevertheless, retain the result within the desired parameters of efficiency and value production.

In short, the present work is bound to have a triple impact: industrial, societal, and theoretical. It has an industrial impact because not only by highlighting the points in the production process, where the resource is overused unnecessarily (sobriety pools), but also by proposing a critical path for dealing with these points, the project will enable the swift achievement of sobriety objectives, in terms of resource consumption, not limited to energy. It will not only make it possible to claim accelerated carbon neutrality, but also to rethink the indicators in terms of the rationality of resource use, thus, resolving the missing link between carbon neutrality and productive processes [62], which is probably at the root of the reluctance to act of the last few decades, given that appetite and the promise of growth were so mutually opposed. Thus, the model guarantees, at worst, the neutrality of exploitation on the productive processes of companies (“sobriety by design”), and at best, the improvement of margins. Once the exogenous slag has been removed as a pretext for inaction (the supposedly deleterious impact of imperative decarbonization efforts on the economy [12]), the only thing that will remain is the real will of economic players, who will no longer have any reason to delay their march towards carbon neutrality and should, on the contrary, accelerate it, since this would be economically beneficial for them.

This ambition is made realistic by the fact that the model and its proof of concept are based on the advanced analysis of industrial and economic processes, thus supported by the existence in the accounting of the principle of “equi-proportionality”, which implies that the performance of tasks of the same nature implies the consumption of the same proportion of resources, so that a principle demonstrated for one process will de facto apply to all processes of the same nature [63].

Thus, the same inductor, i.e., the same unit of work, will not only vary identically but will also consume resources in the same proportions. A variation in one process will have an identical effect on all the identical processes involved. This is called homogeneity of activities. If we show, tomorrow, that every production process has a sobriety potential, all identical activities will have the same characteristics, opening the way to sober growth.

## Supporting information

S1 Table Models variables and notations. **A.** General notation description. **B.** Description of terms used in differential equations.
(PDF)

## References

[1] Balzani V. Saving the planet and the human society: renewable energy, circular economy, sobriety. Substantia. 2019;3(2):9–15.

[2] Gershman SJ, Markman AB, Otto AR. Retrospective revaluation in sequential decision making: a tale of two systems. Journal of Experimental Psychology: General. 2014;143(1):182. doi: 10.1037/a0030844 23230992

[3] Dupuy JP. Le sacrifice et l’envie: le libéralisme aux prises avec la justice sociale. Calmann-Lévy; 2014.

[4] Girard R. Le sacrifice. Éditions de la Bibliothèque nationale de France; 2015.

[5] Kelly MP, Barker M. Why is changing health-related behaviour so difficult? Public health. 2016;136:109–116. doi: 10.1016/j.puhe.2016.03.030 27184821 PMC4931896

[6] Olanrewaju OI, Kineber AF, Chileshe N, Edwards DJ. Modelling the relationship between Building Information Modelling (BIM) implementation barriers, usage and awareness on building project lifecycle. Building and Environment. 2022;207:108556.

[7] De Mesquita BB, Smith A, Siverson RM, Morrow JD. The logic of political survival. MIT press; 2005.

[8] De Vries CE, Hobolt SB. Political entrepreneurs. In: Political Entrepreneurs. Princeton University Press; 2020.

[9] Laîné L. Pour une éthique de la sobriété. Revue d’ethique et de theologie morale. 2018;(HS):117–131.

[10] Gawel JE. Herzberg’s theory of motivation and Maslow’s hierarchy of needs. Practical Assessment, Research, and Evaluation. 1996;5(1):11.

[11] Uribe Saenz S. Constraint, conviction, or convenience? The adoption of environmental standards among palm oil growers in the Colombian llanos.; 2019.

[12] Lamb WF, Mattioli G, Levi S, Roberts JT, Capstick S, Creutzig F, et al. Discourses of climate delay. Global Sustainability. 2020;3.

[13] Weber M. Die protestantische Ethik und der “Geist” des Kapitalismus/[Hauptband]. Die protestantische Ethik und der “Geist” des Kapitalismus. 1905.

[14] Goux JJ, Ascheim K, Garelick R. General economics and postmodern capitalism. Yale French Studies. 1990;(78):206–224.

[15] Foster JB, Holleman H. Weber and the environment: Classical foundations for a postexemptionalist sociology. American Journal of Sociology. 2012;117(6):1625–1673.

[16] Aron R. Les étapes de la pensée sociologique: Montesquieu. Comte Marx Tocqueville Durkheim Pareto Weber Paris: Gallimard. 1976.

[17] Marín-Beltrán I., Demaria F., Ofelio C., Serra L. M., Turiel A., Ripple W. J., et al. (2022). Scientists’ warning against the society of waste. *Science of The Total Environment*, 811, 151359. doi: 10.1016/j.scitotenv.2021.151359 34742963

[18] Ritzer G. McDonaldization: the reader. Pine Forge Press; 2009.

[19] Verdolini E., & Bosetti V. (2017). Environmental policy and the international diffusion of cleaner energy technologies. *Environmental and Resource Economics*, 66(3), 497–536.

[20] LeCun Y., Bengio Y., Hinton G. Deep learning, Nature; 521 (7553), p. 436–444; 2015. doi: 10.1038/nature14539 26017442

[21] Krizhevsky A, Nair V, Hinton G. CIFAR-10 (canadian institute for advanced research). URL http://wwwcstorontoedu/kriz/cifarhtml. 2010;5(4):1.

[22] Stein JG. The Managed and the Managers: Crisis Prevention in the Middle East. In: New Issues in International Crisis Management. Routledge; 2020. p. 171–198.

[23] Ikhlasse H, Benjamin D, Vincent C, Hicham M. Environmental impacts of pre/during and post-lockdown periods on prominent air pollutants in France. Environment, Development and Sustainability. 2021;23(9):14140–14161. doi: 10.1007/s10668-021-01241-2 33519298 PMC7825385

[24] Strubell E, Ganesh A, McCallum A. Energy and policy considerations for deep learning in NLP. arXiv preprint arXiv:190602243. 2019.

[25] Patterson D, Gonzalez J, Hölzle U, Le Q, Liang C, Munguia LM, et al. The carbon footprint of machine learning training will plateau, then shrink. Computer. 2022;55(7):18–28.

[26] Acun B, Lee B, Maeng K, Chakkaravarthy M, Gupta U, Brooks D, et al. A Holistic Approach for Designing Carbon Aware Datacenters. arXiv preprint arXiv:220110036. 2022.

[27] Wu CJ, Raghavendra R, Gupta U, Acun B, Ardalani N, Maeng K, et al. Sustainable AI: Environmental implications, challenges and opportunities. Proceedings of Machine Learning and Systems. 2022;4:795–813.

[28] Tangen S. Demystifying productivity and performance. International Journal of Productivity and performance management. 2005.

[29] Mattei A. (2000). Théorie du consommateur. Dans:, A. Mattei, *Manuel de micro-économie* (pp. 4–69). Genève: Librairie Droz.

[30] Chiappori P. A. (1990). La théorie du consommateur est-elle réfutable?. *Revue économique*, 1001–1025.

[31] Hugon P. (2004). The Boundaries of the Competitive Order and the Market: Global Public Goods and the Commons. Geography, Economy, Society, 6, 265–290. 10.3166/ges.6.265-290.

[32] Jolink A., & Van Daal J. (1998). Gossen’s laws. *History of political economy*, 30(1), 43.

[33] Katzner D. W. (2014). Ordinal utility and the traditional theory of consumer demand. *Real-world Economics Review*, (67), 130–136.

[34] Barnett W. (2003). The modern theory of consumer behavior: Ordinal or cardinal?. *The Quarterly Journal of Austrian Economics*, 6(1), 41–65.

[35] Fustier B., & Rouget B. (1979). *La nouvelle théorie du consommateur est-elle testable*? (Doctoral dissertation, Institut de mathématiques économiques (IME)).

[36] de Lima Salem V., & Picot-Coupey K. (2020). *The adoption of responsible behaviors in food consumption-the case of Brazilian consumers* (Doctoral dissertation, IAE France).

[37] Dufer J., & Moulins J. L. (1989). La relation entre la satisfaction du consommateur et sa fidélité à la marque: un examen critique. *Recherche et Applications en Marketing (French Edition)*, 4(2), 21–36.

[38] Giese JL, Cote JA. Defining consumer satisfaction. Academy of marketing science review. 2000;1(1):1–22.

[39] Leidy NK. Operationalizing Maslow’s theory: Development and testing of the basic need satisfaction inventory. Issues in Mental Health Nursing.1994;15(3):277–295. doi: 10.3109/01612849409009390 7829317

[40] Ray MR, Gupta PP. Activity-based costing. Internal Auditor. 1992;49(6):45–52.

[41] Thomas C, Gervais M, et al. Le problème du regroupement des activités dans la modélisation ABC: une approche possible. Finance Contrôle Stratégie. 2008;11(4):137–170.

[42] Cooper R, Kaplan RS. Profit priorities from activity-based costing. Harvard business review. 1991;69(3):130–135.

[43] Kaplan RS, Cooper R, et al. Cost & effect: using integrated cost systems to drive profitability and performance. Harvard Business Press; 1998.

[44] Menges R. Supporting renewable energy on liberalised markets: green electricity between additionality and consumer sovereignty. Energy Policy. 2003;31(7):583–596.

[45] Aggeri F, et al. Managerial phenomena to the test of standard economic thinking [Les phénomènes gestionnaires à l’épreuve de la pensée économique standard]; 2015.

[46] Boumans M, et al. Built-in justification. IDEAS IN CONTEXT. 1999;52:66–96.

[47] Pardee RL. Motivation Theories of Maslow, Herzberg, McGregor & McClelland. A Literature Review of Selected Theories Dealing with Job Satisfaction and Motivation. 1990.

[48] Terzi S, Bouras A, Dutta D, Garetti M, Kiritsis D, et al. Product lifecycle management-from its history to its new role. International Journal of Product Lifecycle Management. 2010;4(4):360.

[49] Bhattacharya A, Vasant P. Soft-sensing of level of satisfaction in TOC product-mix decision heuristic using robust fuzzy-LP. European Journal ofOperational Research. 2007;177(1):55–70.

[50] Kalybekov Т., Rysbekov K. B., & Soltabayeva S. T. (2018, December). The study of the influence of preparedness of the ore reserves on the planning of underground mining operations. in International Scientific and Technical Internet Conference «Innovative Development of Resource-Saving Technologies of Mineral Mining and Processing (pp. 26–28).

[51] Cui L, Wang Y, Chen W, Wen W, Han MS. Predicting determinants of consumers’ purchase motivation for electric vehicles: An application of Maslow’s hierarchy of needs model. Energy Policy.2021;151:112167.

[52] Huber J. Technological environmental innovations (TEIs) in a chain-analytical and life-cycle-analytical perspective. Journal of Cleaner Production.2008;16(18):1980–1986.

[53] Lester D. Measuring Maslow’s hierarchy of needs. Psychological reports. 2013;113(1):15–17. doi: 10.2466/02.20.pr0.113x16z1 24340796

[54] Crawford K, Joler V. Anatomy of an AI System. Retrieved September. 2018;18:2018.

[55] Donnée impacts environnementaux (2021) LAZARD. https://www.lazard.com/, (accessed as of October 4, 2022).

[56] Bilan GES (2022) ADEME. Available from: https://bilans-ges.ademe.fr/.

[57] Singh DP. Levelized cost of energy, levelized cost of storage, and levelized cost of hydrogen; 2021. Available from: https://www.lazard.com/perspective/levelized-cost-of-energy-levelized-cost-of-storage-and-levelized-cost-of.

[58] Princen T. The logic of sufficiency. Mit Press; 2005.

[59] Crawford K. The atlas of AI: Power, politics, and the planetary costs of artificial intelligence. Yale University Press; 2021.

[60] Krizhevsky A., & Hinton G. (2009). Learning multiple layers of features from tiny images.

[61] He K, Zhang X, Ren S, Sun J. Deep residual learning for image recognition. In: Proceedings of the IEEE Conference on Computer Vision and Pattern Recognition; 2016. p. 770–778.

[62] Peyronel V. Éthique et crise du capitalisme financier anglo-saxon. Revue LISA/LISA e-journal Littératures, Histoire des Idées, Images, Sociétés du Monde Anglophone–Literature, History of Ideas, Images and Societies of the English-speaking World. 2015;13(2).

[63] Sarkar M, Kim S, Jemai J, Ganguly B, Sarkar B. An application of time-dependent holding costs and system reliability in a multi-item sustainable economic energy efficient reliable manufacturing system. Energies. 2019;12(15):2857.

