# Supplementary material for: Making green growth a reality: Reconciling sobriety with stakeholders’ satisfaction
Source: PLoS One. 2023 Aug 23;18(8):e0284487. doi: 10.1371/journal.pone.0284487 (PMC10446189; doi:10.1371/journal.pone.0284487)
Supplement: S1 Table — A. General notation description. B. Description of terms used in differential equations. (PDF) [file pone.0284487.s001.pdf]

S1 A Table. General notation description.

Word/phrase	Abbreviation
Life Cycle Analysis	LCA
activity-based costing method	ABC

S1 B Table. Description of terms used in differential equations

Notation	Description
A	activity
C	cost
T	tasks
I	Inductor (i.e. productive process)
r	resources
P	production of a given good or service
S	Satisfaction function
f, g, z, h	Theoretical Mathematical function
d	data
a_{AI}	AI algorithm
i	evolutive index
m_1 ; m_2	depreciation component; maintenance m_2
e	given energy consumption
i_h	human inputs
z,α,β,ω,μ	respectively the outputs of the processes of extraction, transformation, exploitation, usage, and reuse
$S\ (S_y, S_\omega, S_\beta, S_\alpha, S_z, S_\gamma)$	a multi-objective satisfaction function for each AI process
**Θ**	objective function corresponding to the satisfaction of each process
